# Determinants of Definitive Full Pulpotomy Adoption in Symptomatic Irreversible Pulpitis: A Multivariable Analysis of Dental Trainee Decision-Making

**DOI:** 10.3390/healthcare14101342

**Published:** 2026-05-14

**Authors:** Suzan Cangül, Özkan Adıgüzel, Makbule Taşyürek, Hatice Ortaç

**Affiliations:** 1 Department of Restorative Dentistry, Faculty of Dentistry, Dicle University, Diyarbakir 21280, Turkey; 2 Department of Endodontics, Faculty of Dentistry, Dicle University, Diyarbakir 21280, Turkey; 3 Department of Biostatistics, Faculty of Medicine, Dicle University, Diyarbakir 21280, Turkey

**Keywords:** vital pulp therapy, full pulpotomy, symptomatic irreversible pulpitis, dental trainees, clinical decision-making, endodontic education

## Abstract

Background: Full pulpotomy has gained increasing attention as a conservative treatment option for managing complicated crown fractures and pulp exposures in mature permanent teeth. However, little is known about how undergraduate dental students perceive this treatment approach and which factors influence their willingness to adopt it in clinical practice. Objective: This study aimed to evaluate undergraduate dental students’ knowledge, attitudes, and preferences regarding full pulpotomy and to identify factors associated with willingness to use full pulpotomy as a definitive treatment option. Materials and Methods: A cross-sectional questionnaire-based study was conducted among fourth- and fifth-year dental students. The questionnaire evaluated participants’ treatment preferences, perceived procedural difficulties, preferred pulp capping materials, attitudes toward rubber dam use, perceived barriers to full pulpotomy adoption, and willingness to use full pulpotomy as a definitive treatment. Associations between variables were assessed using chi-square tests and multivariable binary logistic regression analysis. Results: In total, 255 undergraduate dental students participated in the study. Spontaneous pain (69.4%), prolonged pain to heat (50.6%), percussion sensitivity (46.7%), and radiographic findings (43.9%) were the most frequently reported diagnostic criteria for symptomatic irreversible pulpitis. In the standardized clinical scenario, a pulpotomy-based approach was the most preferred treatment strategy (45.1%), followed by single-visit pulpectomy with obturation (28.6%) and pulpectomy with calcium hydroxide dressing (24.7%). MTA was the most preferred pulp capping material (57.3%), followed by Biodentin (12.9%) and calcium hydroxide (8.2%). Overall, 55.7% of participants reported willingness to use full pulpotomy as a definitive treatment option. Clinical year, previous exposure to pulpotomy cases, and confidence in bleeding control were independently associated with willingness to use full pulpotomy. Previous performance of pulpotomy procedures and attitude toward mandatory rubber dam use were independently associated with greater willingness to use full pulpotomy, whereas perceived barriers and uncertainty regarding implementation were negatively associated. Conclusions: In this single-center, questionnaire-based study, undergraduate dental students generally showed a positive attitude toward full pulpotomy; however, acceptance was strongly influenced by practical experience, confidence in procedural protocols, and perceived implementation barriers. These findings may help inform future educational strategies aimed at improving confidence and supporting evidence-based adoption of conservative pulp-preserving approaches.

## 1. Introduction

Vital pulp therapy (VPT) has gained increasing attention in contemporary endodontics as a biologically oriented, minimally invasive alternative to conventional root canal treatment (RCT) in selected cases of symptomatic irreversible pulpitis [1,2]. Historically, mature permanent teeth diagnosed with irreversible pulpitis have been routinely managed with pulpectomy and randomized controlled trials (RCTs) due to their high reported long-term success rates [3]. However, emerging evidence suggests that inflammatory changes in such cases are often confined to the coronal pulp, while radicular pulp tissue may remain viable and capable of repair when appropriately managed [4,5].

Recent clinical investigations have demonstrated favorable outcomes of partial and full pulpotomy in mature permanent teeth diagnosed with symptomatic irreversible pulpitis, particularly when strict clinical protocols are followed [6,7]. The success of VPT depends not only on biomaterials such as mineral trioxide aggregate (MTA) and other calcium silicate-based cements, but also on accurate diagnosis, adequate hemostasis, proper isolation, and careful case selection in accordance with contemporary guidelines [8,9,10].

Despite accumulating clinical evidence and position statements from professional organizations such as the European Society of Endodontology and the American Association of Endodontists [11], full pulpotomy remains underutilized in daily practice [12]. Survey-based studies conducted among general dental practitioners have consistently reported a preference for RCT over pulpotomy in cases of symptomatic irreversible pulpitis [13,14,15]. This discrepancy between scientific evidence and clinical behavior highlights the need to better understand determinants of therapeutic decision-making.

Educational exposure and clinical experience during undergraduate training may play a pivotal role in shaping treatment preferences. Limited hands-on experience, uncertainty regarding long-term prognosis, and lack of confidence in adopting contemporary protocols have been identified as potential barriers to pulpotomy implementation [16,17,18]. Nevertheless, data focusing specifically on dental trainees and the independent predictors of their willingness to adopt full pulpotomy as a definitive treatment remain scarce.

Therefore, the primary aim of the present study was to evaluate dental trainees’ diagnostic approaches and clinical management strategies for symptomatic irreversible pulpitis. The secondary and analytically central objective was to identify independent predictors associated with the willingness to use full pulpotomy as a definitive treatment using multivariable logistic regression analysis.

## 2. Materials and Methods

### 2.1. Study Design and Ethical Approval

This study was designed as a cross-sectional, quantitative survey-based investigation evaluating dental trainees’ diagnostic approaches and clinical management strategies in cases of symptomatic irreversible pulpitis. The study protocol was reviewed and approved by the Ethics Committee of Dicle University Faculty of Dentistry (Approval No: 2025-64; Date: 25 June 2025). All procedures were conducted in accordance with the principles of the Declaration of Helsinki [19]. Participation was voluntary and anonymous with no identifying information such as name or student ID. Participants were informed that non-participation or withdrawal would not affect their academic standing.

### 2.2. Study Population

The target population consisted of fourth- and fifth-year dental students enrolled in the clinical phase of the undergraduate curriculum at Dicle University Faculty of Dentistry (Türkiye). Only students who had completed at least one clinical rotation in endodontics and restorative dentistry were considered eligible for participation. Preclinical students and individuals outside the institution were excluded.

A total of 255 completed questionnaires with valid responses were included in the final analysis (n = 255). Questionnaires with substantial missing data (>20% unanswered items) were excluded prior to analysis. The survey was distributed to 300 eligible fourth- and fifth-year dental students during the study period, and participation was voluntary and anonymous. A total of 255 completed questionnaires were included in the final analysis, corresponding to a response rate of 85.0%.

### 2.3. Sample Size Considerations

A priori power analysis was performed using G*Power 3.1 (Heinrich Heine University, Düsseldorf, Germany). Based on an anticipated medium effect size (w = 0.30), α = 0.05, and statistical power (1 − β) = 0.80, the minimum sample size required for chi-square comparisons between categorical variables was considered adequate. The final sample of 255 participants exceeded the minimum required sample size.

Because the primary analysis of the study involved multivariable logistic regression, model adequacy was additionally evaluated according to the events-per-variable (EPV) principle. Among the 255 participants, 142 reported willingness to consider full pulpotomy as a definitive treatment option. Given the number of predictors included in the final regression model, the events-per-variable ratio exceeded the recommended minimum threshold of 10, indicating that the regression model was adequately powered and unlikely to be substantially overfitted.

### 2.4. Questionnaire Development and Content

The questionnaire consisted of 19 structured items organized into thematic domains including diagnostic criteria, treatment preferences, clinical experience, perceived barriers, and postoperative management strategies (Table 1). Questionnaire items were developed following a review of the contemporary literature on vital pulp therapy, pulpotomy decision-making, and undergraduate dental education. Particular attention was given to current position statements and recommendations published by the European Society of Endodontology regarding management of deep caries and symptomatic irreversible pulpitis. The questionnaire content was reviewed by two endodontists and one restorative dentistry specialist to ensure face validity and relevance to the study objectives.

Before formal distribution, the questionnaire was pilot-tested in a small group of dental trainees (n = 15) to evaluate clarity, readability, and completion time. Minor wording modifications were made following pilot feedback. Data obtained during the pilot phase were not included in the final analysis. The survey was administered electronically via Google Forms (accessed on 15 September 2025, Google LLC, Mountain View, CA, USA). The questionnaire required approximately 8–10 min to complete. To improve response rates, reminders were distributed through official student communication channels and class representatives at two-week intervals.

The clinical scenario included a 22-year-old female patient presenting with nocturnal spontaneous pain associated with tooth 36 and radiographic evidence of deep caries, followed by pulp exposure during caries removal. Because the questionnaire was based on a hypothetical clinical vignette, the primary outcome reflected self-reported willingness to use full pulpotomy rather than actual clinical behavior.

### 2.5. Variables and Operational Definitions

The primary outcome variable was willingness to use full pulpotomy as a definitive treatment option in the presented clinical scenario. Participants selecting responses indicating that they would consider full pulpotomy when appropriate criteria were met were coded as “Yes,” whereas all other responses were coded as “No.” Independent variables included year of study, prior pulpotomy experience, attitudes toward mandatory rubber dam use, perceived importance of hemostasis, treatment preference in the clinical scenario, perceived implementation barriers, and uncertainty regarding barriers. Variables were selected for inclusion in the multivariable logistic regression model based on both univariable screening (*p* < 0.10) and clinical relevance according to the existing literature on decision-making in vital pulp therapy. For categorical predictors with more than two levels, dummy coding was used. In the pulpotomy experience variable, “never performed” was used as the reference category, while “observed only” and “performed” were entered as separate dummy variables. Similarly, for perceived barriers, “no barrier” served as the reference category (Table 2).

### 2.6. Statistical Analysis

All statistical analyses were performed using IBM SPSS Statistics Version 25.0 (IBM Corp., Armonk, NY, USA). Categorical variables were presented as frequency (n) and percentage (%). Comparisons between fourth- and fifth-year students were performed using Pearson’s chi-square test or Fisher–Freeman–Halton test where appropriate. Multivariable binary logistic regression analysis was conducted to identify independent predictors associated with willingness to use full pulpotomy as a definitive treatment option. Odds ratios (ORs) with 95% confidence intervals (CIs) were calculated. Variables with *p* < 0.10 in univariable analyses and variables considered clinically relevant were entered into the final multivariable model. Model fit was assessed using the likelihood ratio test, Hosmer–Lemeshow goodness-of-fit test, Nagelkerke R^2^, and overall classification accuracy. Multicollinearity between predictors was evaluated using variance inflation factor (VIF) and tolerance statistics before model construction. No evidence of problematic multicollinearity was identified among the included variables. A *p*-value < 0.05 was considered statistically significant.

## 3. Results

### 3.1. Participant Characteristics

A total of 255 dental trainees were included in the final analysis. Among them, 117 (45.9%) were fourth-year students and 138 (54.1%) were fifth-year students. The gender distribution was balanced. Detailed demographic characteristics are presented in Table 3.

### 3.2. Diagnostic Criteria and Clinical Exposure

Table 4 summarizes participants’ diagnostic preferences, clinical exposure frequency, treatment selection, preferred pulp capping materials, and willingness to use full pulpotomy as a definitive treatment approach. Among the diagnostic criteria used for symptomatic irreversible pulpitis, spontaneous pain was the most frequently selected finding (69.4%), followed by prolonged pain to heat (50.6%), percussion sensitivity (46.7%), and radiographic findings (43.9%). Electric pulp test response, palpation sensitivity, and prolonged pain to cold were reported less frequently. Regarding clinical exposure, 52.5% of participants reported encountering symptomatic irreversible pulpitis cases at least weekly, whereas 47.5% encountered such cases monthly or less frequently.

In the standardized clinical scenario, the most frequently preferred treatment approach was a pulpotomy-based strategy (45.1%), followed by single-visit pulpectomy with obturation (28.6%) and pulpectomy with calcium hydroxide dressing (24.7%). Only 1.6% of participants selected other treatment options. MTA was the most preferred pulp capping material (57.3%), followed by Biodentin (12.9%) and calcium hydroxide (8.2%). Overall, 55.7% of participants indicated that they would be willing to use full pulpotomy as a definitive treatment option.

### 3.3. Barriers to Full Pulpotomy Adoption

Participants reported several barriers to adopting full pulpotomy as a definitive treatment approach. The most frequently reported barrier was lack of practical experience (52/255, 20.4%), followed by concerns about long-term success (42/255, 16.5%) and limited access to materials (34/255, 13.3%). Insufficient theoretical knowledge was reported by 26 participants (10.2%), whereas 22 (8.6%) reported uncertainty regarding barriers. Lack of confidence was the least frequently reported barrier (9/255, 3.5%). Detailed frequencies are presented in Table 5.

### 3.4. Class-Level Comparisons

Comparisons between fourth- and fifth-year students are shown in Table 6. No statistically significant class-level differences were observed regarding willingness to adopt definitive full pulpotomy (48.7% vs. 57.2%, *p* = 0.153), attitude toward mandatory rubber dam use (50.4% vs. 54.3%, *p* = 0.583), perceived importance of perforation size (88.9% vs. 87.0%, *p* = 0.748), or preference for MTA as the pulp capping material (55.6% vs. 58.7%, *p* = 0.566). In contrast, a statistically significant difference was observed in reported procedural difficulty, with fifth-year students more frequently indicating no procedural difficulty than fourth-year students (35.5% vs. 22.2%, *p* = 0.0129). A significant between-class difference was also observed for hemostasis duration distribution (*p* = 0.0299).

### 3.5. Multivariable Logistic Regression Analysis

Multivariable binary logistic regression analysis was performed to identify independent predictors associated with willingness to use full pulpotomy as a definitive treatment option (Table 7). The overall model was statistically significant (likelihood ratio test, *p* < 0.001) and showed acceptable goodness of fit according to the Hosmer–Lemeshow test (*p* = 0.403). The model explained 24.0% of the variance in the outcome (Nagelkerke R^2^ = 0.240) and achieved an overall classification accuracy of 66.3%. After adjustment for covariates, previously having performed pulpotomy was independently associated with greater willingness to use full pulpotomy as a definitive treatment option (OR 2.51, 95% CI 1.18–5.33, *p* = 0.016). A mandatory attitude toward rubber dam use was also positively associated with willingness (OR 1.78, 95% CI 1.01–3.14, *p* = 0.046).

In contrast, reporting a barrier to implementation was associated with lower willingness to adopt full pulpotomy (OR 0.29, 95% CI 0.15–0.58, *p* = 0.0005). Uncertainty regarding barriers was also negatively associated with willingness (OR 0.06, 95% CI 0.02–0.25, *p* < 0.001). Year of study, observed-only pulpotomy experience, hemostasis requirement, and scenario pulpotomy choice were not independently associated with willingness in the final model (all *p* > 0.05). Complete regression results are presented in Table 7.

Model fit statistics: Hosmer–Lemeshow *p* = 0.403; Nagelkerke R^2^ = 0.240; overall classification accuracy = 66.3%. The subgroup reporting uncertainty regarding barriers was relatively small (n = 22); therefore, the corresponding odds ratio should be interpreted cautiously.

Participants who reported uncertainty regarding implementation barriers constituted a relatively small subgroup (n = 22). Although uncertainty was strongly associated with lower willingness to adopt full pulpotomy, this finding should be interpreted cautiously because of the limited number of participants in this category.

**Figure 1:**
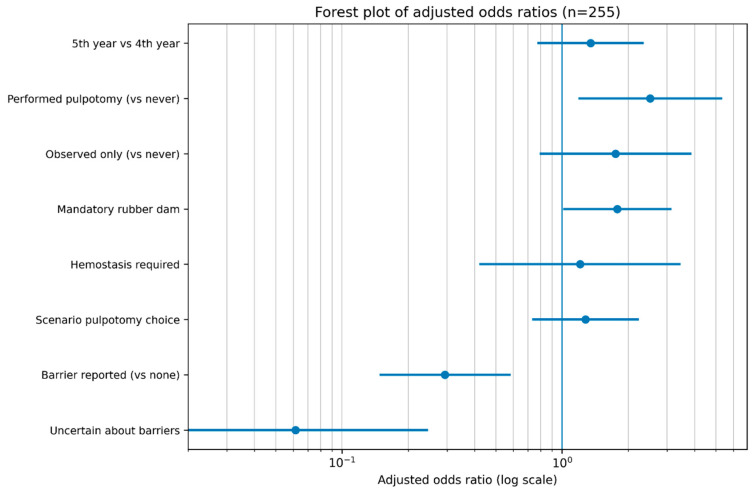
Forest plot of adjusted odds ratios for willingness to adopt full pulpotomy as a definitive treatment option.

The adjusted associations between independent variables and willingness to use full pulpotomy as a definitive treatment are visually presented in Figure 1.

Adjusted odds ratios (ORs) and 95% confidence intervals (CIs) derived from the multivariable logistic regression model are presented. The vertical reference line represents OR = 1.0, indicating no association with the outcome. Values above 1.0 indicate increased willingness to adopt full pulpotomy, whereas values below 1.0 indicate reduced willingness.

## 4. Discussion

The present cross-sectional study evaluated diagnostic approaches and treatment decision-making patterns among dental trainees in the management of symptomatic irreversible pulpitis. Although a considerable proportion of participants reported frequent clinical exposure to symptomatic irreversible pulpitis and selected pulpotomy-based management in the standardized clinical scenario, willingness to adopt full pulpotomy as a definitive treatment remained moderate [20,21]. This finding highlights a persistent gap between theoretical acceptance of biologically based treatment principles and their translation into definitive clinical decision-making.

Contemporary evidence increasingly supports the use of vital pulp therapy (VPT) in mature permanent teeth diagnosed with symptomatic irreversible pulpitis [22,23]. Histopathological and clinical studies have shown that inflammatory changes are often localized to the coronal pulp, while radicular pulp tissue may remain healthy enough to support healing after appropriate intervention [24,25,26]. As a result, recent guidelines from the European Society of Endodontology (ESE) [23] and other professional organizations emphasize that irreversible pulpitis should not automatically be equated with complete pulp removal and root canal treatment. Instead, full pulpotomy may represent a predictable and minimally invasive alternative in carefully selected cases [27,28].

Despite these developments, several studies have shown that clinicians continue to prefer conventional pulpectomy and root canal treatment in cases of symptomatic irreversible pulpitis. Survey-based studies among general dental practitioners in the UK, China, and other countries have demonstrated that concerns regarding prognosis, diagnostic uncertainty, and lack of familiarity with newer VPT protocols frequently limit pulpotomy adoption [13,29,30,31]. The present findings appear consistent with this broader pattern. Even among trainees exposed to current endodontic education, treatment decisions still seem to be influenced by conventional symptom-based frameworks and long-standing assumptions that symptomatic irreversible pulpitis invariably requires root canal treatment.

One of the most important findings of the present study was that prior hands-on pulpotomy experience was independently associated with greater willingness to use full pulpotomy as a definitive treatment option. In contrast, year of study alone was not independently associated with willingness after adjustment for other variables. This distinction is clinically relevant because it suggests that practical exposure may be more important than academic seniority in shaping treatment attitudes [32]. Similar findings have been reported in educational research, where direct procedural experience has been shown to improve confidence, reduce uncertainty, and facilitate the adoption of contemporary treatment concepts [33,34]. Our findings therefore support the idea that supervised clinical exposure to pulpotomy procedures may be essential for improving student confidence and encouraging acceptance of minimally invasive endodontic approaches.

Another important finding was the positive association between mandatory rubber dam use and willingness to adopt definitive full pulpotomy. Rubber dam isolation is considered a core component of contemporary VPT because it ensures aseptic conditions, reduces bacterial contamination, and improves procedural predictability. The ESE position statement on management of deep caries and exposed pulp strongly recommends mandatory rubber dam use during pulpotomy procedures [18,23]. In this context, students who viewed rubber dam use as essential may also have been more likely to embrace evidence-based treatment principles in general. Previous studies have shown that rubber dam use among both dental students and practicing clinicians remains inconsistent, often because of perceived difficulty, limited training, or time constraints [35,36]. The present findings suggest that adherence to structured clinical protocols may be associated with greater openness to conservative pulp-preserving strategies.

Additional recent studies provide a useful framework for interpreting the moderate willingness observed in the present cohort. In a randomized controlled trial, Zhu et al. [21] reported comparable 12-month clinical and radiographic outcomes for full pulpotomy and root canal treatment in mature molars with irreversible pulpitis, while full pulpotomy required less treatment time and cost and produced a greater reduction in pain during the early postoperative period. Similarly, Ather et al. [37] reported pooled pulpotomy success rates exceeding 80% in permanent teeth diagnosed with irreversible pulpitis, supporting the growing evidence base for vital pulp therapy in mature teeth. Nevertheless, these studies also emphasize that successful outcomes depend on appropriate case selection, hemorrhage control, aseptic technique, biomaterial selection, and adequate coronal restoration rather than on the procedure alone.

Recent consensus and cohort evidence further suggest that clinical decision-making in vital pulp therapy should extend beyond a purely symptom-based diagnosis. Zhang et al. [38] emphasized that definitive treatment decisions should incorporate intraoperative reassessment of pulp vitality, particularly hemorrhage control and the clinical appearance of the remaining pulp tissue. Similarly, Asgary et al. [39] demonstrated that symptomatic irreversible pulpitis, apical periodontitis, restoration type, and restoration extent may significantly influence long-term outcomes following vital pulp therapy. These findings may help explain why students appear receptive to pulpotomy conceptually but remain hesitant regarding its definitive implementation, as the procedure requires interpretation of dynamic biological findings and clinical judgment rather than reliance solely on predefined diagnostic categories.

Implementation-related evidence is also highly relevant to the present findings. Colloc et al. reported that conventional root canal treatment remained the predominant management strategy among practitioners in both the USA and UK despite increasing willingness to consider pulpotomy as a definitive treatment option [13]. Likewise, Yi et al. [14] showed that clinicians may continue to prefer root canal treatment because of concerns related to pulp exposure, unfavorable tooth conditions, and fear of persistent postoperative symptoms. An overview of systematic reviews by Chhabra et al. [40] further concluded that although randomized clinical trial evidence increasingly supports pulpotomy as a promising alternative, limitations in the certainty and long-term consistency of the available evidence remain. Collectively, these findings suggest that moderate willingness among trainees should not necessarily be interpreted as resistance to innovation, but rather as a reflection of the ongoing transition in endodontic education and clinical practice, where emerging biological concepts, clinical uncertainty, technical confidence, and evidence interpretation continue to evolve simultaneously.

The negative associations observed for perceived barriers and uncertainty regarding barriers also deserve particular attention. Students who reported barriers to implementation were significantly less likely to support definitive full pulpotomy, while those who were uncertain about barriers demonstrated the lowest willingness overall. These findings suggest that uncertainty itself may represent an important obstacle to treatment adoption. In endodontics, diagnostic ambiguity and uncertainty regarding prognosis have historically contributed to preference for more invasive treatment strategies. Traditional binary classifications of reversible and irreversible pulpitis may oversimplify the biological spectrum of pulpal disease and contribute to hesitation when considering conservative approaches [41,42]. Our findings align with Edwards et al. [29] who identified insufficient training, uncertainty regarding outcomes, and lack of confidence as important barriers to pulpotomy use in primary dental care. Collectively, these findings indicate that educational interventions should focus not only on technical skills, but also on improving students’ confidence in case selection, hemostasis assessment, prognostic interpretation, and evidence-based treatment planning.

The present study also demonstrated that the regression model explained only a moderate proportion of variance in willingness to adopt full pulpotomy. This finding suggests that clinical decision-making is likely influenced by additional cognitive, educational, and behavioral factors not captured in the questionnaire. Variables such as theoretical knowledge level, self-efficacy, perceived procedural competence, tolerance toward clinical uncertainty, and educational environment may all contribute to treatment preferences and professional decision-making processes [43]. Previous educational research has shown that clinical confidence and decision-making behavior are shaped not only by technical exposure, but also by psychological and contextual factors related to training experience and perceived preparedness [44,45].

The study has several strengths. First, it specifically focused on dental trainees, a population that has received relatively limited attention in previous pulpotomy research. Second, the study used a multivariable regression model rather than relying only on descriptive statistics, allowing independent predictors of willingness to be identified more clearly. Third, the response rate was relatively high, and the questionnaire underwent pilot testing and expert review before implementation.

Several limitations should also be acknowledged. First, the cross-sectional design reflects self-reported attitudes at a single point in time and does not allow causal inference. The primary outcome of the study was willingness to use full pulpotomy in a hypothetical clinical scenario rather than actual clinical behavior. Therefore, the results should not be interpreted as evidence that participants would necessarily make the same decisions in real-world practice. Second, the study was conducted in a single institution, which may limit the generalizability of the findings to dental schools with different curricula, levels of clinical exposure, or educational philosophies. Third, the questionnaire did not include several potentially relevant variables associated with clinical decision-making, including knowledge level, self-efficacy, perceived procedural competence, attitudes toward risk, and previous pediatric dentistry experience. In addition, the questionnaire was not based on a previously validated psychometric scale specifically designed to assess attitudes toward full pulpotomy decision-making. Furthermore, knowledge level, self-efficacy, and perceived clinical competence were not directly measured using standardized assessment tools, which may have limited a more comprehensive evaluation of factors influencing treatment preferences. Finally, although the subgroup reporting uncertainty regarding barriers showed a strong negative association with willingness, the relatively small number of participants in this category requires cautious interpretation.

## 5. Conclusions

In this single-center survey, many undergraduate dental students reported a favorable attitude toward the use of full pulpotomy as a definitive treatment option. However, willingness to adopt this approach was associated with previous clinical experience, confidence in procedural protocols, and perceptions regarding treatment barriers. Students who had previously performed pulpotomy procedures and those who considered rubber dam isolation mandatory were more likely to support definitive full pulpotomy.

Lack of practical experience, concerns regarding long-term success, and uncertainty about implementation emerged as important barriers. These findings may support a greater emphasis on supervised clinical exposure, case-based training, and evidence-based education regarding contemporary vital pulp therapy. Strengthening these components may improve student confidence and facilitate wider adoption of conservative pulp-preserving approaches in future clinical practice.

## Figures and Tables

**Table 1:** Structure and thematic distribution of the questionnaire.

Domain	Content Assessed	Example Focus
Demographic Characteristics	Participant profile	Year of study
Diagnostic Criteria	Clinical indicators used for diagnosing symptomatic irreversible pulpitis	Spontaneous pain, thermal response, percussion sensitivity, radiographic findings
Clinical Exposure	Frequency of encountering symptomatic irreversible pulpitis cases	Weekly/monthly frequency
Treatment Preference (Clinical Scenario)	Immediate treatment choice following pulp exposure	Full pulpotomy, pulpectomy, temporary dressing, extraction
Decision-Making Factors	Criteria influencing pulpotomy selection	Perforation size, bleeding control, patient-related factors
Hemostasis Evaluation	Perceived importance and acceptable duration of bleeding control	1–3 min, 3–5 min, 5–10 min
Isolation Protocol	Attitudes toward rubber dam usage	Mandatory, conditional, unnecessary
Material Preference	Preferred pulp capping materials	MTA, Biodentin, calcium hydroxide, others
Clinical Experience	Hands-on experience with pulpotomy	Performed, observed, never performed
Perceived Barriers	Obstacles preventing definitive pulpotomy use	Lack of training, confidence, material access, long-term prognosis concerns
Postoperative Management	Approach to postoperative pain and follow-up	Analgesics, antibiotics, conversion to RCT, radiographic follow-up

**Table 2:** Definition and coding of variables used in multivariable logistic regression analysis.

Variable Type	Variable	Reference Category	Comparison Category/Coding
Outcome	Willingness to use full pulpotomy as a definitive treatment	No/other responses	Yes (if appropriate criteria are met) = 1
Independent variable	Year of study	4th year	5th year = 1
Independent variable	Prior pulpotomy experience	Never performed	Observed only = Dummy 1; Performed = Dummy 2
Independent variable	Mandatory rubber dam attitude	Conditional/unnecessary	Mandatory = 1
Independent variable	Hemostasis necessity	No/uncertain	Yes = 1
Independent variable	Scenario treatment preference	Pulpectomy/other	Full pulpotomy-based approach = 1
Independent variable	Perceived implementation barriers	No barrier reported	Barrier reported = 1
Independent variable	Uncertainty regarding barriers	No	Yes = 1

Reference categories represent the baseline group in logistic regression analyses. For variables with more than two categories, dummy variables were created and entered into the model separately.

**Table 3:** Participant characteristics (n = 255).

Variable		n	%
Year of Study	4th year	117	45.9
	5th year	138	54.1
Gender	Female	128	50.2
	Male	127	49.8

**Table 4:** Diagnostic criteria, clinical exposure, and treatment preferences (n = 255).

Domain	Item	n	%
Diagnostic criteria	Spontaneous pain	177	69.4
	Prolonged pain to heat	129	50.6
	Percussion sensitivity	119	46.7
	Radiographic findings	112	43.9
	Electric pulp test response	73	28.6
	Palpation sensitivity	48	18.8
	Prolonged pain to cold	39	15.3
Exposure frequency	≥Weekly	134	52.5
	Monthly or less	121	47.5
Scenario treatment	Single-visit pulpectomy + obturation	73	28.6
	Pulpectomy + Ca(OH)_2_ dressing	63	24.7
	Pulpotomy-based approach	115	45.1
	Other	4	1.6
Preferred pulp capping material	MTA	146	57.3
	Biodentin	33	12.9
	Calcium hydroxide	21	8.2
	Other/unsure	55	21.6
Outcome	Willingness to use full pulpotomy (Yes)	142	55.7
	Other responses	113	44.3

Participants were allowed to select more than one response only for diagnostic criteria items; therefore, percentages in that subsection do not total 100%.

**Table 5:** Reported barriers to adoption of full pulpotomy.

Barrier	n	%
Lack of practical experience	52	20.4
Concerns about long-term success	42	16.5
Limited access to materials	34	13.3
Insufficient theoretical knowledge	26	10.2
Uncertain/not sure	22	8.6
Lack of confidence	9	3.5

Percentages were calculated based on the total study population (n = 255). Participants were allowed to select more than one barrier.

**Table 6:** Comparison between fourth- and fifth-year students.

Variable	4th Year n (%)	5th Year n (%)	*p*-Value
Willingness to adopt definitive full pulpotomy (Yes)	57 (48.7)	79 (57.2)	0.153
Reported no procedural difficulty	26 (22.2)	49 (35.5)	0.0129
Mandatory rubber dam attitude (mandatory)	59 (50.4)	75 (54.3)	0.583
Perforation size considered important (Yes)	104 (88.9)	120 (87.0)	0.748
Preferred pulp capping material = MTA	65 (55.6)	81 (58.7)	0.566
Hemostasis duration distribution (all categories)			0.0299
Hemostasis duration < 3 min	10 (7.0)	13 (11.5)	
Hemostasis duration 3–5 min	83 (58.5)	58 (51.3)	
Hemostasis duration > 5 min	34 (23.9)	26 (23.0)	
Bleeding amount more important/unsure	15 (10.6)	16 (14.2)	

Chi-square test or Fisher–Freeman–Halton test as appropriate. Hemostasis duration categories were defined as <3 min, 3–5 min, >5 min, and bleeding amount more important/unsure.

**Table 7:** Multivariable logistic regression for willingness to use full pulpotomy as a definitive treatment (n = 255).

Predictor	OR	95% CI	*p*-Value
5th year vs. 4th year	1.35	0.77–2.36	0.295
Performed pulpotomy (vs. never)	2.51	1.18–5.33	0.016
Observed only (vs. never)	1.68	0.77–3.64	0.190
Mandatory rubber dam	1.78	1.01–3.14	0.046
Hemostasis required	1.39	0.48–4.00	0.546
Scenario pulpotomy choice	1.59	0.90–2.80	0.107
Barrier reported (vs. none)	0.29	0.15–0.58	0.0005
Uncertain about barriers	0.06	0.02–0.25	<0.001

## Data Availability

The data presented in this study are available on request from the corresponding author due to patient privacy and ethical reasons.
